# Human exposure to anopheline mosquitoes occurs primarily indoors, even for users of insecticide-treated nets in Luangwa Valley, South-east Zambia

**DOI:** 10.1186/1756-3305-5-101

**Published:** 2012-05-30

**Authors:** Aklilu Seyoum, Chadwick H Sikaala, Javan Chanda, Dingani Chinula, Alex J Ntamatungiro, Moonga Hawela, John M Miller, Tanya L Russell, Olivier J T Briët, Gerry F Killeen

**Affiliations:** 1 Liverpool School of Tropical Medicine, Vector Group, Pembroke Place, Liverpool, L3 5QA, UK; 2 National Malaria Control Centre, PO Box 32509, Lusaka, Zambia; 3 Ifakara Health Institute, Biomedical and Environmental Thematic Group, Kiko Avenue, PO Box 78373, Dar es Salaam, Tanzania; 4 PATH Malaria Control and Evaluation Partnership in Africa (MACEPA), National Malaria Control Centre, Lusaka, Zambia; 5 James Cook University, Faculty of Medicine, Health and Molecular Sciences, Cairns, Australia; 6 Swiss Tropical and Public Health Institute, Department of Public Health and Epidemiology, Basel, Switzerland; 7 University of Basel, Basel, Switzerland

**Keywords:** *Anopheles funestus*, *Anopheles quadriannulatus*, Behaviour, Zambia

## Abstract

**Background:**

Current front line malaria vector control methods such as indoor residual spraying (IRS) and long-lasting insecticidal nets (LLINs), rely upon the preference of many primary vectors to feed and/or rest inside human habitations where they can be targeted with domestically-applied insecticidal products. We studied the human biting behaviour of the malaria vector *Anopheles funestus* Giles and the potential malaria vector *Anopheles quadriannulatus* Theobald in Luangwa valley, south-east Zambia.

**Methods:**

Mosquitoes were collected by human landing catch in blocks of houses with either combined use of deltamethrin-based IRS and LLINs or LLINs alone. Human behaviour data were collected to estimate how much exposure to mosquito bites indoors and outdoors occurred at various times of the night for LLIN users and non-users.

**Results:**

*Anopheles funestus* and *An. quadriannulatus* did not show preference to bite either indoors or outdoors: the proportions [95% confidence interval] caught indoors were 0.586 [0.303, 0.821] and 0.624 [0.324, 0.852], respectively. However, the overwhelming majority of both species were caught at times when most people are indoors. The proportion of mosquitoes caught at a time when most people are indoors were 0.981 [0.881, 0.997] and 0.897 [0.731, 0.965], respectively, so the proportion of human exposure to both species occuring indoors was high for individuals lacking LLINs (*An. funestus*: 0.983 and *An. quadriannulatus*: 0.970, respectively). While LLIN users were better protected, more than half of their exposure was nevertheless estimated to occur indoors (*An. funestus*: 0.570 and *An. quadriannulatus*: 0.584).

**Conclusions:**

The proportion of human exposure to both *An. funestus* and *An. quadriannulatus* occuring indoors was high in the area and hence both species might be responsive to further peri-domestic measures if these mosquitoes are susceptible to insecticidal products.

## Background

Long-lasting insecticidal nets (LLINs) and indoor residual spraying (IRS) are the two principal methods of reducing malaria transmission in Africa [1-6] and both rely on killing and/or deterring mosquitoes attempting to feed and/or rest in houses [7,8]. The behaviour of various malaria vectors in relation to vector control interventions has been extensively reviewed [7]. Some populations of *Anopheles arabiensis* are known to bite extensively outdoors in the early evening [9] while most people are outdoors and awake, and some studies suggest that vectors may adopt such behaviours in response to high coverage of IRS or ITNs [10]. More recently, altered feeding patterns of vector mosquitoes resulting in a shift of human exposure from occurring indoors during hours when most people are asleep, toward occurring outdoors in the evenings and mornings, have been reported following increased use of LLINs and IRS in Tanzania [11], Equatorial Guinea [12] and the Solomon Islands [13].

In Zambia, both LLINs and IRS have been dramatically scaled up in recent years [14], and the official national target is to achieve 100% coverage of all populations at risk with locally appropriate interventions by 2012 [15]. The country was an early recipient of donor support to scale-up malaria control efforts through funding from the Global Fund, the President Malaria Initiative, World Bank Booster Program and Bill & Melinda Gates Foundation [16].

In Zambia, IRS was introduced in 2003, and as of the 2011–2012 transmission season, IRS services are offered at varying levels of coverage in all 72 districts. Many areas of endemic transmission in Africa, including Zambia, have conducted extensive LLIN distribution campaigns which have dramatically increased bed net coverage in previously unprotected populations. Zambia has distributed nets both through mass campaigns and through antenatal care (ANC) clinics with the aim to achieve high coverage [17]. However, even given high levels of net ownership, usage levels of nets in many of these areas is unsatisfactory [18,19]. Furthermore, recent studies have shown that the current ITN distribution strategies, that miss households occupied by the elderly and those without children or ANC access, cannot reach 100% coverage, even if the number of nets should be sufficient to reach this level [17].

Both the personal and community-level impact of IRS and ITNs are entirely dependent upon mosquitoes entering houses, [9,20,21] and thus understanding the behaviour of malaria vectors in relation to vector control interventions is fundamental; importantly, behaviour is highly variable across the spectrum of vector species [7]. Despite the massive investment in the use of LLINs and IRS as front-line vector control measures across the tropics, there are only a few estimates of the proportion of malaria transmission occuring indoors and can be directly prevented by these measures [9,11,13,22-24]. Zambia is primarily reliant upon these measures for national-scale malaria vector control, and estimates of the proportion of human exposure occurring indoors are important to understand the success of malaria control efforts.

In this paper, we provide a description of the behaviour of malaria vectors in the Luangwa valley in south-east Zambia, to better understand the efficacy of intra-domiciliary vector control interventions in terms of the proportion of human exposure to malaria transmission that occurs indoors and can potentially be prevented. These studies were also undertaken to establish a baseline assessment to enable future evaluation of how sustained use of vector control interventions will affect underlying important malaria vector behaviours such as their peak feeding times and preferences for feeding either indoors or outdoors.

## Methods

### Study area

**Figure 1:**
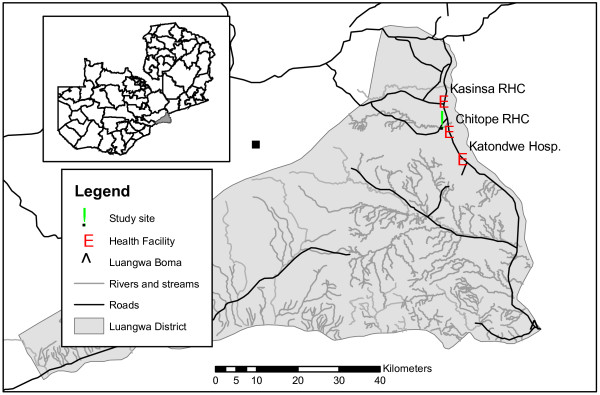
Location of the study area, Luangwa district, South-east Zambia.

The study was conducted in Chisobe and Nyamumba villages, located in the northern part of the Luangwa district, south-east Zambia (Figure 1) where mosquito breeding habitats associated with a spring-fed stream exist throughout the year and malaria transmission is perennial. Fishing from the Luangwa River is the main socioeconomic activity, and subsistence farming is also commonly practiced. Goats and chickens are kept by many households, as well as cattle amongst a smaller minority. Between the villages, a perennial stream flows from Chakolwe escarpment to Luangwa River and creates numerous breeding sites for mosquitoes. Rainfall is strongly seasonal: the main rainy season occurs from November through March with the average annual amount of rainfall ranging between 600 mm and 1400 mm. The village population at Chisobe was approximately 250 and that of Nyamumba was 100, with a total of about 55 households. Chisobe village is located along the only main road in Luangwa beside a small perennial stream with Nyamumba village located approximately 3 km to the west beside a hot spring which forms the source of the same stream. The area is endemic for malaria but the species of vectors involved had not formally been identified before this study.

At the time of this study, LLINs were the only widely used form of vector control for which supplementation with IRS was being planned by the National Malaria Control Centre. Luangwa district was one of the initial pilot districts for mass distribution of LLINs, beginning in late 2005, with additional nets provided in 2006 for mass distribution and antenatal care clients since 2007. Two rounds of mass distribution of LLINs were carried out in 2008 and 2009 in the two villages where this study was carried out, and the number of nets distributed per household depended on family size. Households with family sizes of one to three, four to five and above six received one, two and three LLINs, respectively, and this was followed by interpersonal communication activities where the community health workers (CHWs) assisted the community through regular sensitization regarding the proper use of nets on a continuous basis. Furthermore, additional campaigns that distributed new LLINs to households which had no functional LLINs were carried out by the CHWs in 2010 and 2011.

Indoor residual spraying was introduced to the district in late 2010 as part of the national plans for scaling up IRS. Initially, IRS was targeted in the southern half of Luangwa district and subsequent spraying seasons plan to further introduce IRS to areas of the district as funding improves. IRS activities at the study sites described in this study were introduced specifically for the purposes of research.

### Study design

Mosquitoes were collected using human landing catches indoors and outdoors in both the dry (September-October 2009) and wet seasons (February–March 2010) for a total of 60 nights to determine the feeding behaviour of the malaria vectors and proportion of indoor exposure in the area. This was carried out for 30 consecutive nights during the dry season and another 30 nights during the wet season.

In each village, two blocks of three houses each were selected for mosquito sampling based on logistical convenience, acceptance of the residents and a minimum spacing of about 100 m between blocks. All the houses have LLINs and were utilized by all members of the household during the study period. Following the LLIN distribution and promotion campaigns that have occurred in the district [17,18], by far the most commonly used net product is PermaNet 2.0®, a factory-treated rectangular polyethylene net containing deltamethrin at a target dose of 55 mg/m^2^ as the active ingredient. In each village, one of the block was randomly chosen and all houses in this block were sprayed three days before commencement of the experiments with K-Othrine® WG 250 formulation of deltamethrin insecticide (Bayer Environmental Science), applied to the inner surfaces of the walls and roofs at a dosage of 20 mg.m^-2^ of the active ingredient.

Mosquitoes were collected for twelve hours each night from 19 h in the evening up to 7 h in the morning. Catching was conducted for 45 min each hour, allowing 15 min rest. In order to estimate the biting rate for a full hour, the number of mosquitoes of a given species caught per hour was divided by 0.75. Samples were kept in labelled paper cups for each hourly catch. Each night, mosquitoes were sampled by human landing catches (HLC) indoors and outdoors (about 2 m away from the house) of one house of each of the four blocks as part of an evaluation of a variety of mosquito-trapping methods (Sikaala et al., Unpublished). The three houses in each block were each sampled once over three consecutive nights of rotation of HLC through the block as described in further elsewhere (Sikaala et al., Unpublished). Mosquitoes were first identified to sex and to species morphologically [25] and all female anophelines were preserved in Eppendorf tubes with desiccating silica gel. Samples of *An. gambiae* sensu lato and *An. funestus* s.l. sibling species were further identified by polymerase chain reaction [26].

The proportion of time residents spent outdoors and indoors was estimated from answers to questionnaires during a cross-sectional household survey in April 2010 in Luangwa district, in which people indicated the time they usually went indoors and when they went to the bed as well as when they arose in the morning and when they left their houses [22-24,27]. The coverage and utilization of nets were also determined through the household survey using a questionnaire developed by the MEASURE DHS + programme and adopted and recommended for use by the RBM MERG Task force on household surveys [28].

### Data analysis

The interventions used (LLINs and IRS) have different modes of providing vector control. LLINs can confer both personal and community-wide protection. Whereas IRS, confers negligible direct personal protection to occupants of sprayed houses. This is because this method for delivering insecticides to houses can kill mosquitoes before or after they have fed, and can even kill mosquitoes that the occupants would never have been exposed to because they entered the house to rest but not feed. The concept that *de facto* levels of personal protection only apply to the fraction of human exposure which occurs indoors is therefore specifically applied here only for LLINs.

Conventional indices of behavioural patterns for malaria vectors can substantively underestimate the potential of LLINs because they ignore the behaviour of the human host in whether they are indoors or outdoors [29]. The average proportion of human exposure to bites of a given vector population which occur indoors in the absence of any protective measure such as an LLINs (*π*_*i*_), was calculated by weighting the mean indoor (*B*_*i*_) and outdoor (*B*_*o*_) biting rates for each hour of the night (*t*) by the proportion of humans reporting to have been indoors (*I*) and outdoors (1 − *I*), respectively, at that time:

(1) $$\pi_{i}=\sum_{\text{t}=0}^{23}\left[{\text{B}}_{\text{i},\text{t}}{\text{I}}_{\text{t}}\right]/\sum_{\text{t}=0\;}^{23}\left[{\text{B}}_{\text{i},\text{t}}{\text{I}}_{\text{t}}+{\text{B}}_{\text{o},\text{t}}\left(1-{\text{I}}_{\text{t}}\right)\right]$$

This quantity describes the maximum possible degree of personal protection any exclusively indoor measure can provide [9,11,13]. In order to simplify the formal notation required to describe such functions, we define a night as starting at some time between the nightly periods of malaria vector activity so that each of these are captured within a single, continuous 24 h cycle. However, mosquito collections were carried out only in 12 h of night periods from *t* = 1 *up to t* = 12 when most *Anopheles* mosquitoes are biting humans. The most common regional language of East Africa offers a particularly convenient convention for recording time in relation to the behaviour of locally-relevant *Anopheles*: the 12 h *kiSwahili* clock starts at 6 am and 6 pm so that 7 am and 7 pm (7 and 19 h on the standard 24 h clock) are described as *saa moja*, meaning one o’clock. Here we extend directly from this concept, which is also applied in several languages in the horn of Africa, to introduce a sequence of 24 h that begins at 18 h on the conventional 24 h clock so that *t* = 0 corresponds to the period from 18.00 to 19.00 h, *t* = 1 begins at 1 o’clock on the Swahili clock and corresponds to 19.00 to 20.00 h, continuing through *t* = 23 for the period from 17.00 to 18.00 h.

Direct personal protection by using an LLIN is usually only accrued when a person is not only indoors, but also sleeping or trying to sleep in a protected space which the net is hung over. The household survey questionnaire data collected in this study enables calculation of this slightly more specific maximum fraction of human exposure which an LLIN can realistically confer direct personal protection against. The proportion of human exposure to bites of a given vector population which occurs when residents are both indoors and sleeping or trying to sleep (*π*_*s*_) was calculated similarly to (*π*_*i*_), using the same denominator estimate of total indoor and outdoor exposure (Eq. 1) but a numerator which is the sum of the products of the mean indoor (*B*_*i*_) biting rates and the estimated proportions of humans reporting to have gone to bed to sleep (*S*) for each hour of the night (*t*):

(2) $$\pi_{s}=\sum_{\text{t}=0}^{23}\left[{\text{B}}_{\text{i},\text{t}}{\text{S}}_{\text{t}}\right]/\sum_{\text{t}=0}^{23}\left[{\text{B}}_{\text{i},\text{t}}{\text{I}}_{\text{t}}+{\text{B}}_{\text{o},\text{t}}\left(1-{\text{I}}_{\text{t}}\right)\right]$$

Given that IRS is being actively considered as a supplementary intervention option in this setting, we introduce a new parameter that can be estimated from these data that reflects the proportion of exposure of LLIN users which occurs indoors. The proportion of residual human exposure for users of nets which occurs indoors (*π*_*i*,*n*_) was calculated by adjusting the indoor biting rates for the sleeping fraction of the population in proportion to the mean of published estimates [30] for the personal protection (*ρ*) provided by the specific LLIN product predominantly used in this setting:

(3) $$\pi_{i,n}=\sum_{\text{t}=0}^{23}\left[{\text{B}}_{\text{i},\text{t}}\;\left({\text{S}}_{\text{t}}\;\left(1-\rho \right)+\left({\text{I}}_{\text{t}}-{\text{S}}_{\text{t}}\right)\right)\right]/\sum_{\text{t}=0}^{23}\left[{\text{B}}_{\text{i},\text{t}}\left({\text{S}}_{\text{t}}\left(1-\rho \right)+\left({\text{I}}_{\text{t}}-{\text{S}}_{\text{t}}\right)\right)+{\text{B}}_{\text{o},\text{t}}\left(1-{\text{I}}_{\text{t}}\right)\right]$$

No appropriate experimental hut data is available from any Zambian setting at the time of this study, so we set a parameter value of 93.7% feeding inhibition, as personal protection (*ρ* = 0.937) is often referred to, reflecting the mean of studies in neighbouring Tanzania [30,31].

The proportion of exposure to mosquito bites of unprotected individuals which occurs indoors (Equation 1) was also estimated in a more simplified binomial fashion, in which the night is split into distinct periods during which all exposure is assumed to occur either entirely indoors or entirely outdoors, so that it could be analyzed statistically with logistic regression models [11,13]. Then the nightly interval that is considered as normal sleeping time is defined as beginning at the first (*f*) and last (*l*) hour when the majority of people were indoors (*I*_*t*_ > 0.5) because they reported that they had already entered their houses and had not yet left for the day (*f* ≤ *t* ≤ *l*). The remaining periods of the night, before (*t* < *f*) and after (*t* > *l*) this interval, correspond to periods when most people are outdoors (*I*_*t*_ < 0.5). Correspondingly, the proportion of human exposure for non-users of LLINs that occurs indoors was therefore approximately calculated by dividing the number of mosquitoes caught (*N*_*i*_) indoors during the period that most people are indoors by itself plus the number of mosquitoes caught outdoors (*N*_*o*_) outside of that period [9,11,13]:

(4) $$\pi_{i}=\sum_{\text{t}=\text{f}}^{\text{l}}\left[{\text{N}}_{\text{i},\text{t}}\right]/\left(\sum_{\text{t}=0}^{\text{f}-1}\left[{\text{N}}_{\text{o},\text{t}}\right]+\sum_{\text{t}=\text{f}}^{\text{l}}\left[{\text{N}}_{\text{i},\text{t}}\right]+\sum_{\text{t}=\text{l}+1}^{23}\left[{\text{N}}_{\text{o},\text{t}}\right]\right)$$

Note that no equivalent binomial calculation for *π*_*s*_ could be made because this parameter requires subdivision of human exposure into three, rather than two, behavioural compartments. In order to more clearly interpret the *π*_*i*_ and *π*_*s*_ estimates obtained, the two following underlying determinants of these outcomes were also calculated. The propensity of vectors to feed indoors is reflected in the proportion of mosquitoes caught that were captured indoors (*P*_*i*_):

(5) $$P_{i}=\sum_{\text{t}=0}^{23}\left[{\text{N}}_{\text{i},\text{t}}\right]/\sum_{\text{t}=0}^{23}\left[{\text{N}}_{\text{i},\text{t}}+{\text{N}}_{\text{o},\text{t}}\right]$$

The propensity of vectors to feed at times when people are indoors is reflected in the proportion of all mosquitoes caught that were captured during hours when the majority of people were indoors (*P*_*fl*_):

(6) $$P_{\mathrm{fl}}=\sum_{\text{t}=\text{f}}^{\text{l}}\left[{\text{N}}_{\text{i},\text{t}}+{\text{N}}_{\text{o},\text{t}}\right]/\sum_{\text{t}=0}^{23}\left[{\text{N}}_{\text{i},\text{t}}+{\text{N}}_{\text{o},\text{t}}\right]$$

The models fitted to these binomial dependent variables using R statistical software version 2.14.1 supplemented with the lattice and lme4 packages included date (d.f. = 60) and households (d.f. = 12) as random effects and village (Chisobe versus Nyamumba), season (wet versus dry) and treatment (IRS plus LLIN versus LLINS alone) as fixed effects (d.f. = 1 in all cases). These indicators of propensity of vectors to feed indoors (*P*_*i*_) and during the night time hours predominantly spent indoors by humans *P*_*fl*_ (referred to as nocturnality [11] or nocturnal biting [13] in previous publications) were tested for vector preference (*P*_*i*_ or *P*_*fl*_ ≠ 0.5) in terms of the significance of the differences of these estimates from the null hypothesis (*P*_*i*_ or *P*_*fl*_ = 0.5).

### Ethical considerations

Both oral and written consent were obtained from all participants involved with human landing catches. Malaria prophylaxis with deltaprim (the recommended chemoprophylaxis in Zambia) was also provided on a weekly basis throughout the study period. The study protocol was approved by the national ethics committee based at the University of Zambia and the Ethics Committee of Liverpool School of Tropical Medicine (Reference numbers FWA00000338 and 09.60, respectively).

## Results

### Species composition, vector feeding activity and human exposure patterns

A total of 7756 female anopheline mosquitoes were caught by the human landing catches in a total of 240 catcher-nights. Of those anophelines caught, 8.3 and 43.6% were morphologically identified as *An. gambiae* s.l., and *An. funestus* s.l., respectively. The remaining 48% were mainly *An. coustaniAn. rufipesAn. pretoriensis*, and *An. squamosus*. Out of the total of 1179 successfully amplified samples of *An. gambiae* s.l., 95.2% (n = 1122) were identified as *An. quadriannulatus*, while the remainder were *An. arabiensis* (3.9%; n = 46) and *An. gambiae* sensu stricto (0.9%; n = 11). The *An. gambiae* sibling species complex was thus strongly dominated by *An. quadriannulatus*, which are generally believed to be highly zoophilic and play a negligible role for malaria transmission [32]. Out of 440 successfully amplified samples of *An. funestus* s.l., 72.2% (n = 317) were *An. funestus* s.s.*,* while the remainder were zoophilic members of the group namely, *An. rivulorum* (16.2%; n = 71), *An. parensis* (9.8%; n = 43), *An. vaneedeni* (1.4%; n = 7) and *An. leesoni* (0.5%; n = 2).

**Figure 2:**
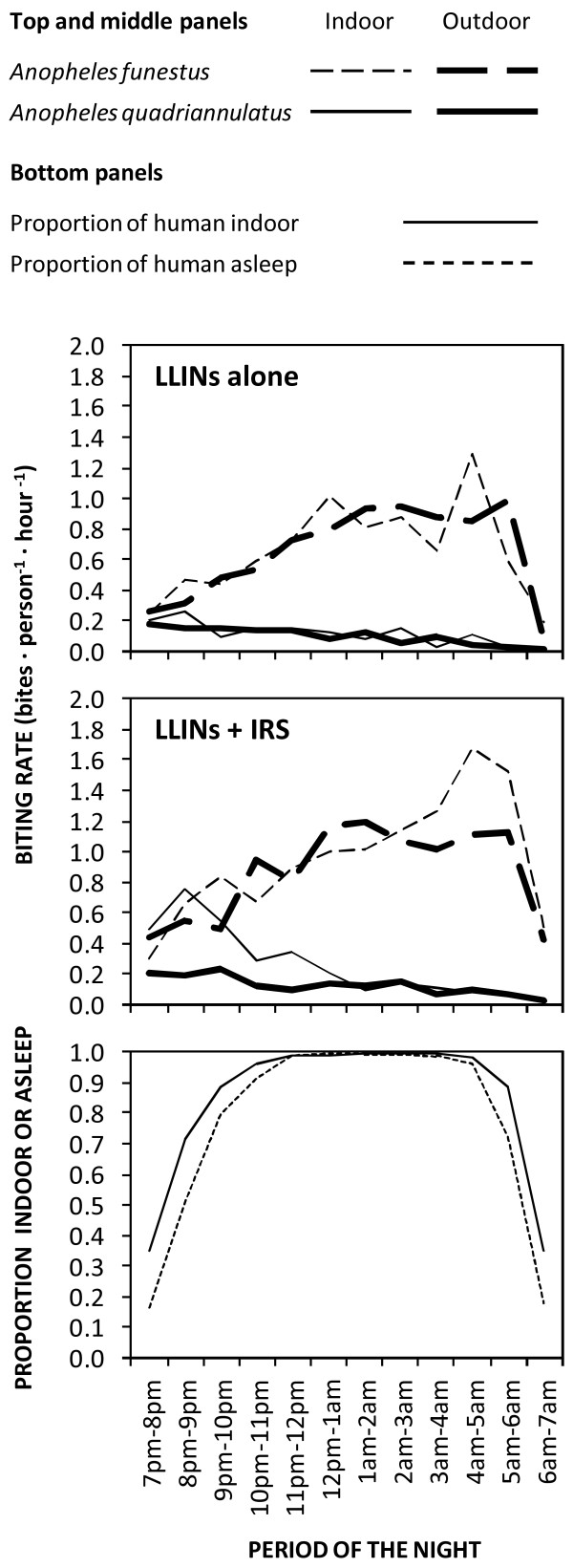
**The observed human biting patterns of* An funestus* and* An. quadriannulatus* in Luangwa Valley, south east Zambia in blocks with either only LLINs (top) or with both LLINs and IRS (middle) with the human movement indoor/asleep (bottom).**

**Figure 3:**
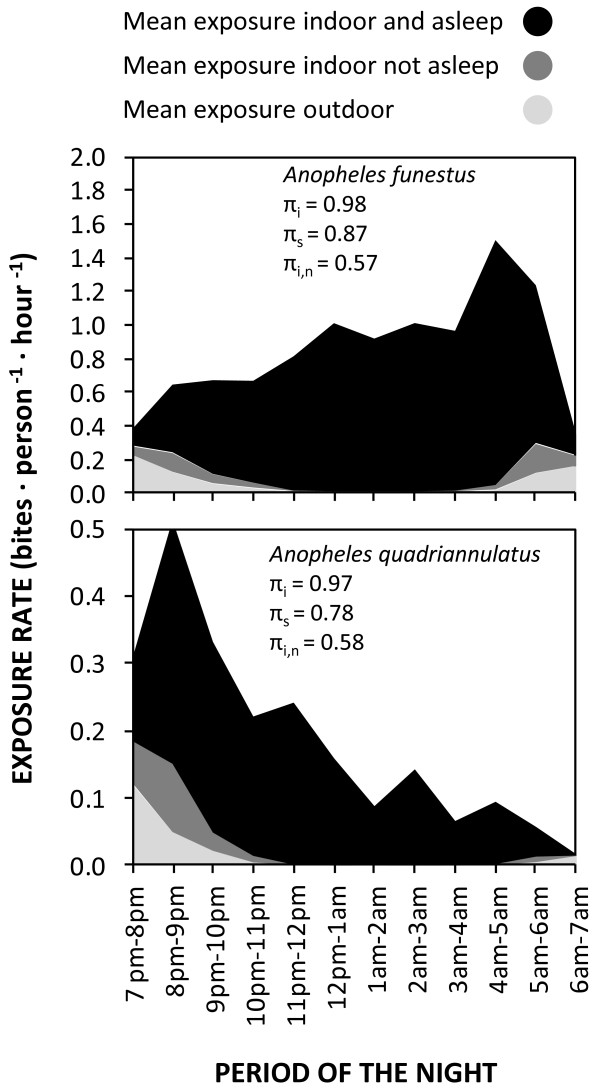
**Estimated mean exposure indoor and outdoor for* Anopheles funestus* (top) and* Anopheles quadriannulatus*(bottom) in Luangwa valley south east Zambia.**

Neither *An. funestus* s.l. nor *An. quadriannulatus* exhibited any clear preference for feeding inside houses in this study area (Table 1), with almost equal proportions of *An. funestus* caught indoors (*P*_*i*_) and outdoors (1- *P*_*i*_). As illustrated in Figure 2, the peak of biting activity by *An. quadriannulatus* coincided with the average time that most people enter their houses to go to sleep at around 20 h (*t* = 2). Consistent with historical reports [25], the biting activity of *An. funestus* was consistently high throughout the late night between midnight to just before sunrise (24 to 6 h) when the vast majority of people were indoors and asleep (Figure 2). While these two anopheline species might superficially appear to have different biting activity patterns (Figure 2), their overall propensity to feed at times when most people are indoors (*P*_*fl*_) was high for both species: the vast majority of *Anopheles* mosquitoes were caught at times when most people are indoors (Table 1). The estimated proportion of exposure to both *An. funestus* s.s. and *An. quadriannulatus* that occurs indoors was therefore high for individuals lacking LLINs (*π*_*i*_) despite the lack of any apparent preference for indoor feeding by either mosquito species (Table 1 and Figure 2). Interestingly, none of the three binomial measures of vector behaviour (*P*_*i*_ and *P*_*fl*_) and vector-host behavioral interaction (*π*_*i*_) were affected by IRS treatment for any of the *Anopheles* taxa surveyed (p ≥ 0.05), indicating that this residual pyrethroid formultion has negligible impact upon house entry and host-seeking behaviours. Slightly over half of the residual exposure ITN users experience occurs indoors (*π*_*i,n*_ = 0.570 and 0.583, for *An. funestus* and *An. quadriannulatus*, respectively) (Figure 3). According to the 2010 cross sectional household survey, 66% of children under five years old and 64.8% of pregnant women slept under an ITN in Luangwa district at that time, and these were the population subgroups where ITN use was highest. .

**Table 1:** **Proportion of anopheline mosquitoes caught indoors, proportion when most people are indoors, and proportion of human exposure occuring indoors for non-ITN and IRS users in Luangwa valley, south-east Zambia**^a^

**Mosquito species**	**Proportion caught indoors (P**_**i**_)^**b**^		**Proportion caught when most people are indoors (P**_**fl**_)^**c**^		**Proportion of human exposure occurring indoors (π**_**i**_)^**d**^	
	**Estimate [95% CI]**	**p**	**Estimate [95% CI]**	**p**	**Estimate [95% CI]**	**P**
*An. funestus*	0.586 (0.303, 0.821)	0.565	0.981 (0.881, 0.997)	<0.001	0.983 (0.845, 0.998)	<0.001
*An. quadriannulatus*	0.624 (0.324, 0.852)	0.425	0.897 (0.731, 0.965)	<0.001	0.970 (0.811, 0.996)	<0.001
Other anophelines	0.467 (0.233, 0.717)	0.809	0.913 (0.762, 0.972)	<0.001	0.855 (0.674, 0.966)	0.002

^a^ All models include date and household as random effects and season, village and treatment as fixed effects. For *An. quadriannulatus*, proportion of human exposure occurring indoors (*π*_*i*_) was significantly affected by village (p = 0.0224) and the proportion caught indoors (*P*_*i*_) was affected by village and season, p = 0.0177 and 0.0238, respectively. Otherwise, none of the estimates for the proportion of human exposure indoors (*π*_*i*_), proportion caught indoors (*P*_*i*_) and proportion caught when most people are indoors (*P*_*fl*_) were not significantly affected by treatment, season or village (P > 0.05) for *An. funestus*, *An. quadriannulatus* or other *anophelines.*

^b^ As described in Equation 5 and associated text in the Methods section.

^c^ As described in Equation 6 and associated text in the Methods section.

^d^ As described in Equation 4 and associated text in the Methods section

## Discussion

In the absence of personal protection by an LLIN, human exposure contact with both *An. quadriannulatus* and *An. funestus* overwhelmingly occurred indoors in this setting (*π*_*i*_ > 0.9), simply because that is where most people spend their time when these species are most active (*P*_*fl*_ = 0.9). Contrary to commonly held views about *An. funestus* and most members of the *An. gambiae* complex, in this setting neither species exhibited any detectable preference for biting indoors during the study period so the bulk of human exposure occurs indoors at night because this is where human and mosquito activities coincide. This analysis of a Zambian vectorial system adds further weight to recent [9] and historical [23,24] suggestions that evaluations of vector behaviour should separately and quantitatively summarize mosquito preference for feeding indoors and for feeding at times when humans are indoors.

*Anopheles quadriannulatus* is readily susceptible to malaria infection [33] and does occasionally feed upon humans [34], as documented here in south-east Zambia (Figure 2), but it is widely regarded as being as preferentially zoophagic and therefore of negligible importance to malaria transmission [35-37]. The large number of *An. quadriannulatus* collected by human landing catches in this setting supports the view that host choice is very plastic in this species and that they may well feed on humans in some settings. Torr and colleagues [34] suggest that *An. quadriannulatus* has no specific preference for animals and also feeds on humans, but simply responds in proportion to overall body mass, which is consistent with how anthropophagic species respond to individual humans [38]. While *Anopheles quadriannulatus* readily feed on humans in Luangwa, no sporozoite-infected specimen was identified in this setting and we could find no report of such an occurrence in the literature, so it is most likely of negligible importance to malaria transmission.

The predominant malaria vector species caught in this study area was *An. funestus* s.s., which is commonly regarded as one of the most efficient malaria vector species in the world because of its very high degree of anthropophagy and endophagy [25,32,39-42]. *Anopheles funestus* s.l. in the study area showed no clear preference for feeding indoors or outdoors (Figure 2 and Table 1). Nevertheless, estimates of the proportion of human exposure which is preventable through the personal protection arising from LLINs use are as high as any published estimate [9,11] in the current study, and indicate that both IRS and LLINs remain excellent options for malaria vector control and should achieve their full potential personal and communal protective effects in this setting. This is contrary to the recent evidence from other parts of the world that suggests that behavioural changes of malaria vectors toward feeding predominantly in the early part of the evenings and outdoors render LLINs less protective [7,10-13,43,44].

The estimate that more than half of all transmission, presumably mostly by *An. funestus* s.s., accrued by LLIN users occurs indoors (*π*_*i,n*_ > 0.5) suggests that supplementing LLINs with IRS might well achieve incremental impact upon malaria transmission in this setting. For effective combined implementation of IRS and LLINs, the insecticide of choice for IRS should ideally have a different mode of action from those used on LLINs to minimize risk of rapid selection of resistance traits in vector populations [45]. As slightly less than half of residual transmission for LLIN users occurs outdoors, there is a limit to how much incremental control can be achieved by complementing LLINs with further indoor vector control using IRS, regardless of how efficacious the product used for the latter is. Therefore, measures which protect against outdoor exposure or which suppress mosquito breeding [46] may well be required to supplement even a combination of LLINs and IRS targetted at endophagic vectors to go beyond malaria control and achieve local elimination in this setting.

Both recent [12,13] and historical [47] reports indicate that as LLINS and IRS are scaled up, previously ignored outdoor-biting mosquitoes become culpable for a greater proportion of malaria transmission. In most cases, this increased proportion of transmission exposure that occurs outdoors appears to be arising from selective suppression of indoor-biting vectors by ITNs [48-50] or IRS [47,51]. Evidence of heritable changes in behavioural traits within single species also seem to be associated with selective suppression of specific molecular [12] and chromosomal [52,53] forms. Perhaps the main limitation of this study is that, while most residents slept under an LLIN, coverage remains incomplete, falls short of the Roll Back Malaria targets [54] and was only achieved relatively recently. It is therefore possible that further selective suppression of the endophagic vectors may occur as LLIN usage rates are increased and sustained. If this plausible scenario were to occur, the majority of human exposure might well occur outdoors so options such as repellents [55], insecticide-treated clothes [56] and insecticide-treated cattle [57] might be higher priority, possibly rendering supplementary IRS redundant. It is therefore important to distinguish between two quite different scenarios: 1) Full coverage of LLINs has achieved maximal suppression of endophagic and endophilic vectors and could be genuinely supplemented with complementary measures, and 2) Partial LLIN coverage with incompletely suppressed indoor transmission so that IRS essentially fills gaps in coverage and therefore partially substitutes for LLINs as a means to tackle persistent indoor transmission. We therefore suggest that national systems for monitoring intervention coverage, malaria risk, and insecticide resistance should now be supplemented by field surveys of vector population composition and the relevant behaviours they exhibit.

This study has some additional minor limitations which can be improved upon in future studies. The estimates for potential protective efficacy of the indoor interventions against bites may be slightly overestimated because mosquitoes were not sampled between 6 pm and 7 pm when some low levels of biting activity can occur. This seems particularly true for *An. quadriannulatus*, for which the peak biting hours are the early part of the evening, but this is not of significant concern because this species appears to have a negligible role in malaria transmission. Another important factor that needs due future consideration is the potential for seasonal variation in the outdoor sleeping behaviour of human populations. In this specific case, household survey data on the movement of people collected during the wet season could underestimate the proportion of people outdoors. Despite these limitations, this study does represent a useful baseline, with which future observations of vector population composition or behaviour can be compared. It also provides a clear example of how distinct, complementary estimates of the location-specific feeding behaviours which underpin where and when humans are exposed to them can be used to inform, plan and rationalize integrated vector management packages.

## Conclusions

The estimated proportion of human exposure to *An. funestus* s.l. occuring indoors was high for individuals lacking LLINs in this setting. However, for the majority of humans that use LLINs, slightly more than half of residual exposure to bites occurs indoors. Therefore, the malaria vectors in the area might be responsive to further peri-domestic control measures if the mosquitoes are susceptible to insecticidal products.

## Competing interests

The authors declare that they have no competing interests.

## Authors’ contributions

AS designed and implemented mosquito sampling protocol in collaboration with other authors. He also analysed the data and drafted the manuscript in consultation of the other authors, CHS implemented mosquito sampling protocol and reviewed the manuscript, JC and DC participated in the implementation of the protocol, AJN and MH participated in the molecular analysis of mosquito samples, JMM contributed the human movement data during the cross-sectional survey, TLR was involved in the design of the field mosquito sampling protocol, OB significantly contributed to the methodology and the manuscript, GFK supervised the design of experiments, analysed the data and drafted the manuscript with the first author. All authors read and approved the manuscript.
